# A Novel Frameshift Variant in the SPAST Gene Causing Hereditary Spastic Paraplegia in a Bulgarian–Turkish Family

**DOI:** 10.3390/neurolint17100167

**Published:** 2025-10-11

**Authors:** Mariya Levkova, Mihael Tsalta-Mladenov, Ara Kaprelyan

**Affiliations:** 1 Department of Medical Genetics, Medical University Varna, Marin Drinov Str. 55, 9000 Varna, Bulgaria; 2 Laboratory of Medical Genetics, St. Marina Hospital, Hristo Smirnenski Blv. 1, 9000 Varna, Bulgaria; 3 Department of Neurology and Neuroscience, Medical University Varna, Marin Drinov Str. 55, 9000 Varna, Bulgaria

**Keywords:** hereditary spastic paraplegia, SPAST gene, whole exome sequencing, genotype–phenotype correlation, underrepresented populations

## Abstract

Background: Hereditary spastic paraplegia (HSP) is a clinically and genetically heterogeneous group of neurodegenerative disorders characterized by progressive lower-limb spasticity and weakness. SPAST mutations are the most common cause of autosomal dominant HSP (SPG4). However, many pathogenic SPAST variants are unique and genetic data from underrepresented communities remain limited. Methods: Whole-exome sequencing (WES) was performed on the index patient with HSP. Variant annotation tools included Ensembl VEP, LOFTEE, CADD, SIFT, PolyPhen-2, MutationTaster, and SpliceAI. Variant interpretation followed ACMG/AMP guidelines. Clinical evaluation and family history supported phenotypic correlation and segregation. Results: A novel heterozygous frameshift variant in SPAST (c.339delG; p.Glu114Serfs*47) was identified. The variant was predicted to cause nonsense-mediated decay, resulting in loss of the microtubule-interacting and AAA ATPase domains of spastin. It was absent from population databases (gnomAD, TOPMed, 1000 Genomes) and public variant repositories (ClinVar, HGMD). The variant segregated with disease in two affected siblings and could be classified as likely pathogenic. Conclusions: This novel SPAST frameshift variant expands the mutational spectrum of SPG4-HSP and highlights the importance of including isolated or minority communities in genomic research to improve variant interpretation.

## 1. Introduction

Hereditary spastic paraplegia (HSP) encompasses a collection of rare inherited neurological conditions, with an estimated worldwide prevalence of 3.6 cases per 100,000 people [1]. Historically referred to as Strümpell–Lorrain disease, the disorder is named after two 19th-century physicians who independently outlined its defining characteristics [2,3]. Clinically, HSP is primarily marked by gradually worsening spasticity and weakness in the lower limbs, often leading to a stiff gait. Additional signs may include increased muscle tone at rest, exaggerated tendon reflexes, ankle clonus, abnormal reflexes, and in some cases, disturbances in bladder and bowel control, reduced vibration sensation, clawfoot deformity, and spinal curvature [4,5].

The onset of HSP symptoms can occur across a broad age range, from early infancy to adulthood. Based on age of onset, the condition is classified into two forms: early-onset (before the age of 35) and classical (onset after age 35) [4]. Clinically, HSP is also divided into two main subtypes—pure and complex [4]. The pure form demonstrates the classical clinical presentation, described above, while the complex form is characterized by additional neurological or systemic features, including cognitive decline, muscle atrophy, ataxia, seizures, and developmental impairments [4].

From a genetic standpoint, HSP can follow autosomal dominant (AD), autosomal recessive (AR), or X-linked inheritance patterns. To date, there are over 80 genetic loci and 79 genes associated with various HSP subtypes (Supplementary Table S1) [4]. The autosomal dominant form is the most prevalent, accounting for about 80% of cases [6]. Among these, SPG4 is the most frequently encountered subtype [4]. It is linked to mutations in the SPAST gene (MIM: 182601), which encodes the protein spastin. Mutations in this gene are responsible for around 40% of autosomal dominant cases and about 20% of sporadic cases [4].

In this report, we describe a novel frameshift variant in the SPAST gene identified in two siblings affected by hereditary spastic paraplegia from a Turkish–Bulgarian family. To our knowledge, this variant has not been previously reported in the literature or in publicly available variant databases. Its identification expands the known mutational spectrum of SPAST and contributes to a better understanding of genotype–phenotype correlations in HSP.

## 2. Case Description

### 2.1. Patient 1 (Proband)

**Figure 1:**
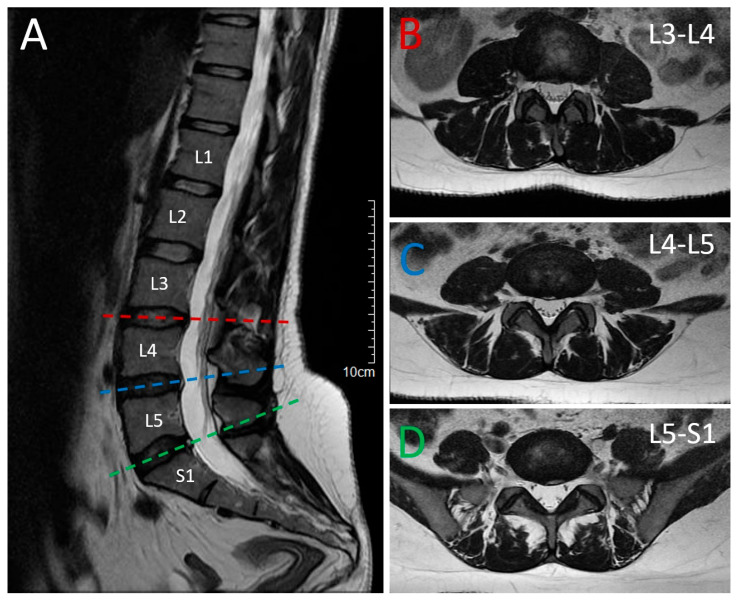
Magnetic Resonance Imaging (MRI) of the lumbar spine. (**A**) T2-weighted sagittal spine magnetic resonance imaging (MRI)showing degenerative changes in the lumbar spine with multilevel bulging of the intervertebral discs, osteochondrosis. Horizontal lines indicate the corresponding levels in the axial views. (**B**–**D**) T2-weighted axial spine MRI at different levels presenting the degenerative changes and intervertebral disc protrusions L3–L4, L4–L5 and L5–S1.

A 41-year-old woman presented with progressive gait disturbance and stiffness of the lower limbs, which developed insidiously over a period of 4 years. She reported dragging of the right leg, feeling of stiffness, and reduced walking endurance. Initial evaluation by a rheumatologist focused on persistent low back pain and the stiffness, raising suspicion of ankylosing spondylitis, which was excluded after appropriate imaging and laboratory evaluation. The initial neurological examination revealed clinical presentation of a pure form of spastic paraparesis, characterized by mild weakness in both lower limbs (MRC grade 4/5), spasticity in both lower limbs, hyperreflexia with polykinetic responses, bilateral sustained clonus, positive Babinski sign bilaterally, and a spastic–paretic gait. The remaining neurological examination, including cranial nerves, upper limb strength, coordination, and sensation, was unremarkable. Lumbar MRI showed mild multilevel disc protrusions without evidence of myelopathy (Figure 1).

Electromyography (EMG) showed signs of a left-sided L4–L5 radiculopathy. Neurosurgical consultation concluded that there was no indication for surgical intervention. Given the progressive pyramidal signs and positive family history, hereditary spastic paraplegia was suspected, and blood samples were collected for genetic testing. Motor evoked potentials (MEPs) and somatosensory evoked potentials (SSEPs) were not performed due to technical limitations.

**Figure 2:**
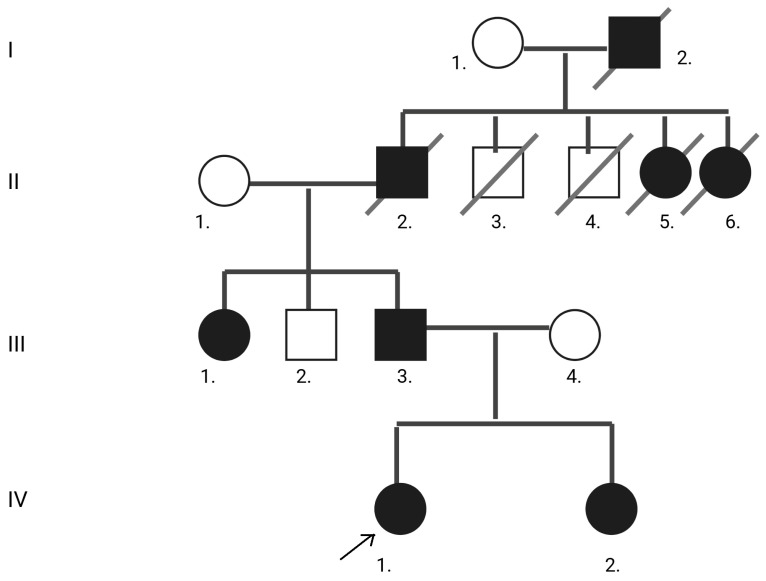
Pedigree tree of the affected sisters. The proband is indicated with an arrow. Distant relatives marked as affected are based on family history only.

The patient reported multiple paternal relatives with similar complaints, although none had a confirmed diagnosis (Figure 2).

### 2.2. Patient 2 (Affected Sibling)

The patient’s 35-year-old sister reported a long-standing history of low back pain dating back to adolescence, with radicular pain into the right lower limb. She also reported urinal incontinence, for which she was also consulted by a gynecologist. Over the previous several months, she had developed cervical spine pain and worsening gait with a sense of instability. The neurological examination revealed weakness in the right upper and lower limbs (MRC grade 4/5), spasticity of the lower limbs, hyperreflexia in the lower extremities with a sustained right-sided foot clonus and hypoesthesia in the C6–C7 dermatomes on the right and L5 on the left. The remaining neurological status was unremarkable.

**Figure 3:**
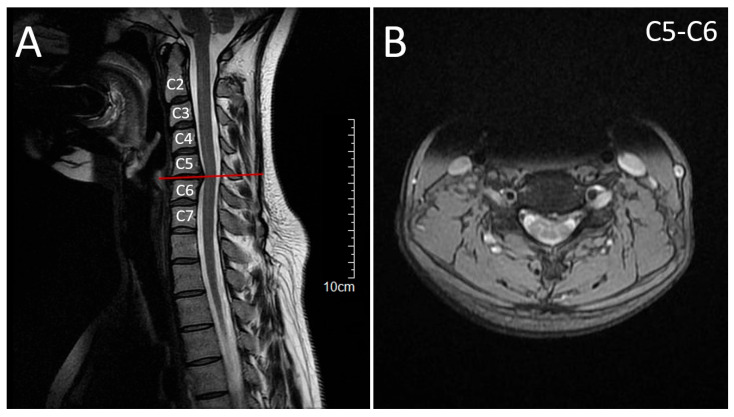
Magnetic Resonance Imaging (MRI) of the cervical spine. (**A**) T2-weighted sagittal spine magnetic resonance imaging (MRI) demonstrating a C5–C6 intervertebral disc herniation with compression of the dural sac but no signal changes suggestive of myelopathy. The horizontal reference line indicates the axial level shown in (**B**). (**B**) T2-weighted axial spine MRI at C5–C6 confirming the disc herniation with dural sac compression, without evidence of myelopathic changes.

Cervical MRI showed a C5–C6 disc herniation contacting the spinal cord but without myelopathy, and lumbar CT demonstrated mild disc bulging (Figure 3).

EMG and nerve conduction studies showed C5–C8 radiculopathy and axonal degeneration of the right median nerve, consistent with chronic sensorimotor peripheral nerve involvement. Based on the symptomatic cervical disc herniation, the patient was referred for neurosurgical management. However, based on the positive results of her sister she was referred to genetic counseling for targeted genetic analysis.

### 2.3. Molecular Genetic Findings

Whole-exome sequencing was performed on an Illumina next-generation sequencing platform at Blueprint Genetics (Espoo, Finland) on peripheral-blood genomic DNA from the proband. Data were aligned to the GRCh37 (hg19) reference genome and analyzed under Blueprint’s validated pipeline. Variant annotation (Ensembl VEP v104 with LOFTEE, CADD v1.7, SIFT, PolyPhen-2, and SpliceAI plugins) identified a single clearly disease-relevant change in SPAST: NM_014946.4:c.339delG, predicted at the protein level as p.(Glu114Serfs*47) (chr2:32,284,628delG; hg19 coordinates) (Table 1) [7,8,9,10,11,12].

The one-base deletion occurs in the first coding exon of both major SPAST transcripts (ENST00000315285.3 and ENST00000345662.1), shifts the reading frame at codon 114, and introduces a premature termination codon 47 residues downstream. Because the new stop lies more than 50 nucleotides upstream of the final exon–exon junction, the mutant transcript is expected to undergo nonsense-mediated decay (NMD). Any residual truncated product would terminate before the MIT domain (aa 116–197) and would completely lack the AAA ATPase domain (aa 342–616) that is essential for spastin’s microtubule-severing function, providing a strong mechanistic basis for loss of function.

In silico pathogenicity metrics support this interpretation. LOFTEE flagged the variant as high-confidence loss-of-function [7]. SIFT and PolyPhen-2 both returned damaging calls, and MutationTaster classified it as disease-causing [10,11,13]. A CADD PHRED score in the high-deleterious range (>20) is typical for comparable early truncating SPAST variants; although a direct retrieval was unsuccessful at the time of analysis, the combination of transcript position, domain loss, and established gene mechanism renders the precise numeric score less critical to classification [8]. SpliceAI showed no evidence that the deletion creates or disrupts splice motifs beyond the primary coding consequence [12]. Gene-level constraint metrics further reinforce a haploinsufficiency model: SPAST has a low LOEUF (≈0.22) and a high predicted probability of haploinsufficiency (pHaplo ≈ 0.975), consistent with intolerance to protein-truncating variation [7].

The variant was absent from gnomAD v4, 1000 Genomes, and TOPMed population datasets at the time of review, supporting rarity [7,14,15]. It was also not listed in ClinVar, HGMD (public), or locus-specific SPAST variant databases, indicating that it is novel [16,17,18]. Clinically, the proband presented with progressive spastic paraparesis characteristic of autosomal dominant Hereditary Spastic Paraplegia (HSP; SPG4). Sanger sequencing confirmed that the affected sister carried the same heterozygous variant. Although parental DNA was not available, the father has a highly similar clinical picture. He developed progressive gait disturbance and spasticity beginning in his mid-40s, further supporting segregation within the family. The other affected family members were also reported to have gait difficulties in adulthood, though they were not formally examined. In light of the newly identified SPAST variant in our patients, we believe that the other affected relative on paternal side was likely affected by the same disorder.

Applying American College of Medical Genetics/Association for Molecular Pathology (ACMG/AMP) guidelines [19], and following the 2024 ACGS Best Practice recommendations, the variant fulfills the following criteria: PVS1 (frameshift → predicted NMD in SPAST, where LOF is an established mechanism), PM2 (absent from population databases), and PP1 (segregation in two affected siblings, father clinically affected). Although multiple in silico tools predict a deleterious effect, PP3 is not combined with PVS1 for LOF variants under the current guidelines. Taken together (PVS1 + PM2 + PP1), the variant is classified as likely pathogenic, and this explains the HSP phenotype in this family. This appears to be the first published clinical description of this specific allele and therefore expands the known mutational spectrum of SPAST.

## 3. Discussion

Our case report describes a previously unreported SPAST frameshift variant, c.339delG; p.(Glu114Serfs*47), in a Bulgarian–Turkish family, a genetically isolated and understudied population whose community-specific variants are largely absent from reference datasets [20,21]. SPAST loss-of-function alleles account for roughly 40% of autosomal-dominant hereditary spastic paraplegia pedigrees and 20% of sporadic cases [4], yet even within this well-studied gene, more than half of pathogenic variants in ClinVar are single-family or “private” [16]. Balkan series illustrate the point: in two Turkish cohorts, 56% of SPAST mutations were unique to a single pedigree, implying serial founder effects rather than a few recurrent alleles [22].

The c.339delG variant extends that spectrum, and because it truncates spastin before the MIT and AAA domains, its molecular mechanism almost certainly involves nonsense-mediated decay (NMD) and haploinsufficiency—the same path seen in most adult-onset SPG4 cases. Large cohort studies confirm that age at onset in SPAST-HSP (SPG4) is strikingly variable but follows a bimodal distribution [23]. The early-childhood peak is driven mainly by missense substitutions, whereas loss-of-function variants, like in our case, that trigger nonsense-mediated decay, populate the broader adult-onset peak between the 3rd and 5th decades [23]. A Portuguese population study that modelled age of onset in 239 AD-HSP carriers found a significantly younger onset for missense versus truncating SPAST alleles (mean difference ≈ 7 years; *p* = 0.015), illustrating the role of the specific genetic variant [24].

Given that the proband’s variant c.339delG; p.Glu114Serfs*47 is an early-exon frameshift predicted to undergo NMD, age at onset is expected in adulthood or late adolescence (median ~30 years), although penetrance is age-dependent and exceptions (both asymptomatic elders and very-early cases) are well documented [23]. The phenotype in our family, adult-onset, pure spastic paraparesis with slow progression, is concordant with other truncating *SPAST* variants, reinforcing the strong correlation between loss-of-function variants and pure HSP forms [4].

More than 80% of SPAST pathogenic variants ultimately reduce functional spastin dosage (haploinsufficiency). These LOF alleles, especially when they truncate the protein before the MIT and AAA domains as here, almost always produce the ‘pure’ phenotype with the typical progressive lower-limb spasticity and hyperreflexia [4]. Like in our case, MRI and neurophysiology are usually normal or show only subtle corticospinal changes in pure SPG4 [4].

A diagnostic challenge in HSP is its overlap with leukodystrophies, primary lateral sclerosis, X-linked adrenomyeloneuropathy, hereditary ataxias, and amyotrophic lateral sclerosis [4]. Moreover, twenty-eight of eighty-one HSP subtypes carry alternative phenotypes in OMIM making the differential diagnosis even more vast [6,25]. In genetically isolated populations, where founder mutations may reside in genes not covered by commercial panels, narrow testing risks false negatives [22]. Whole-exome sequencing therefore in our case offered a comprehensive, cost-effective first-tier approach: it delivered a diagnosis in a single step, avoided multiple sequential panels, and preserved data for future re-analysis.

Management for SPG4 remains symptomatic—physiotherapy, oral antispastic agents, botulinum toxin, anticholinergics for bladder dysfunction [26,27]. Evidence for disease-modifying therapy is still preliminary. Microtubule-stabilising agents, tubulin-acetylation enhancers, and gene-replacement strategies have shown promise in cell and animal models [28], while high-throughput screens have identified small molecules that up-regulate the intact SPAST allele or modulate downstream pathways such as axonal transport [26,29]. By documenting yet another loss-of-function allele, our study strengthens the rationale that restoring spastin dosage should remain a prime therapeutic goal [26].

Segregation data were incomplete as parental DNA was unavailable, limiting formal co-segregation statistics to a single heterozygous sister. Nevertheless, genetic counseling of the children of the affected individuals is recommended. Early molecular genetic identification allows proactive physiotherapy, spasticity management, and counselling on family planning options, including pre-implantation genetic testing [22].

Finally, this report highlights a broader equity issue. Under-sampling of minorities skews variant databases and hampers confident interpretation of novel alleles [21]. Collaborative Balkan registries and targeted outreach can help close that gap, but integration of diverse genomes into global genetic datasets is indispensable. Each novel variant added, from c.339delG in the Bulgarian–Turkish community to rare alleles in other isolated groups, incrementally improves diagnostic accuracy, guides personalised care, and lays the foundation for inclusive precision medicine in hereditary spastic paraplegia. Documenting variants from underrepresented populations is essential, as many remain missing from global variant databases, limiting equitable diagnostic yield. Submission of rare variants to databases such as ClinVar facilitates future recognition and comparison in both diagnostic and research settings.

Several limitations warrant acknowledgement. Functional studies confirming transcript decay or protein loss were not feasible, and the small pedigree restricts statistical power. Nonetheless, the variant fulfils ACMG PVS1 + PM2 criteria [19], is absent from gnomAD [7], and targets a mutational hotspot with strong prior evidence of haplo-insufficiency. Future work should focus on including patient-derived induced pluripotent stem cells or spastin-deficient axon models, thus aiming to clarify the cellular impact of early-exon truncations and identify modifier pathways that influence expressivity.

## 4. Conclusions

We report a novel SPAST frameshift variant, causing autosomal dominant hereditary spastic paraplegia in a Bulgarian–Turkish family. Its identification in an underrepresented population underscores the need for broader inclusion in genomic research, as unique or private variants may be missed in commonly studied cohorts. Documenting such variants contributes to a better understanding of HSP genetics and informs future therapeutic development.

## Figures and Tables

**Table 1:** Characteristics of the reported variant.

Category	Annotation
Gene	*SPAST* (*SPG4* locus)
Transcript	NM_014946.4/ENST00000315285.3 (canonical)
Genomic coordinate (GRCh37)	chr2:32 284 628 delG
cDNA change	c.339delG
Protein change	p.(Glu114Serfs*47)
Consequence	Frameshift variant → premature stop; predicted nonsense-mediated decay
Domain impact	Truncates before MIT domain; AAA ATPase domain absent
CADD (v1.7)	34.9 (highly deleterious; comparable frameshifts)
Constraint metrics	LOEUF = 0.22; pHaplo = 0.975
Population frequency	Not present in gnomAD v4, 1000 Genomes, or TOPMed
ACMG classification	The following ACMG criteria were met: ✓ PVS1: Null variant (frameshift) in a gene where LoF is known mechanism; frameshift → NMD → haploinsufficiency ✓ PM2: Absent from controls; not present in gnomAD ✓ PP1: Co-segregation with disease, present in both affected sisters PVS1 (very strong) + PM2 (moderate) + PP1 (supporting) → Likely pathogenic
Clinical note	Novel variant; segregates with disease in two affected siblings; consistent with autosomal-dominant HSP

## Data Availability

The original contributions presented in this study are included in the article/Supplementary Material. Further inquiries can be directed to the corresponding author.

## Supplementary Materials

The following supporting information can be downloaded at: https://www.mdpi.com/article/10.3390/neurolint17100167/s1, Table S1: Review of the available in OMIM genes and the matching type of hereditary spastic paraplegia, together with relevant clinical information for the specific type [25].

## Abbreviations

The following abbreviations are used in this manuscript:

aa	amino acid(s)
ACMG	American College of Medical Genetics and Genomics
AD	autosomal dominant
AD-HSP	autosomal dominant hereditary spastic paraplegia
AMP	Association for Molecular Pathology
AR	autosomal recessive
chr	chromosome
CT	computed tomography
DNA	deoxyribonucleic acid
EMG	electromyography
gnomAD	Genome Aggregation Database
GRCh37	Genome Reference Consortium human build 37
hg19	human genome 19
HGMD	Human Gene Mutation Database
HSP	hereditary spastic paraplegia
LOF	loss of function
MIM	Mendelian Inheritance in Man
MIT	Microtubule Interacting and Trafficking (domain)
MRC	Muscle strength grade
MRI	magnetic resonance imaging
NMD	nonsense-mediated decay
OMIM	Online Mendelian Inheritance in Man
pHaplo	predicted probability of haploinsufficiency
PM2	Pathogenic Moderate 2
PolyPhen-2	Polymorphism Phenotyping v2
PP1	Pathogenic Supporting 1
PP3	Pathogenic Supporting 3
PVS1	Pathogenic Very Strong 1
SPG4	spastic paraplegia type 4
SpliceAI	splicing-effect prediction tool
TOPMed	Trans-Omics for Precision Medicine
VEP	Variant Effect Predictor
