# Supplementary material for: A Novel Frameshift Variant in the SPAST Gene Causing Hereditary Spastic Paraplegia in a Bulgarian–Turkish Family
Source: Neurol Int. 2025 Oct 11;17(10):167. doi: 10.3390/neurolint17100167 (PMC12566801; doi:10.3390/neurolint17100167)
Supplement: Supplementary file 1 [file neurolint-17-00167-s001.zip › neurolint-3863745-supplementary.pdf]

Location	Phenotype	Inheritance	Phenotype MIM number	Gene/ Locus	Typical onset	Phenotype2	Key clinical features (headline)	Protein function	Sub-cellular localisation	MRI hallmark	Additional symptoms
1p36.13	Spastic paraplegia 78	AR	617225	ATP13A2	Juvenile / adult	C	Kufor-Rakeb: parkinsonism + HSP	Lysosomal P5-ATPase	Lysosome membrane	Diffuse atrophy + BG iron	Dementia, supranuclear gaze palsy
1p34.1	Spastic paraplegia 83	AR	619027	HPDL	Teens	P	Juvenile pure HSP	4-HPPD-like enzyme (unknown)	Mitochondria	Normal / mild	Mild myalgia, dysarthria
1p31.1-p21.1	Spastic paraplegia 29	AD	609727	SPG29	Teens	P	Rare adolescent pure HSP	–	–	–	–
1p13.3	?Spastic paraplegia 63	AR	615686	AMPD2	Infancy	C	Spasticity + PCH-10 features	AMP deaminase 2	Cytoplasm	Pontocerebellar hypoplasia	Severe DD, seizures
1p13.2	Spastic paraplegia 47	AR	614066	AP4B1	Childhood	C	AP-4 deficiency: non-ambulatory spasticity + ID	Adaptor-protein-4 β-subunit	Golgi / endosome	Thin CC, cerebral atrophy	Seizures, strabismus
1q32.1	Spastic paraplegia 23	AR	270750	DSTYK	Childhood–teens	C	Spastic paraplegia with urinary tract malformations	Dual-serine/threonine kinase regulating ERK & FGF signalling	Cytosol & plasma membrane	Often normal; occasional thin CC	LUT anomalies, skin pigmentation defects

1q42.13	?Spastic paraplegia 44	AR	613206	GJC2	Childhood–teens	C	Pelizaeus-Merzbacher-like hypomyelinating HSP	Connexin-47 gap-junction channel	Oligodendrocyte myelin sheath	Diffuse hypomyelination	Nystagmus, ataxia, cognitive delay
1q42.13	?Spastic paraplegia 74	AR	616451	IBA57	Childhood	C	Spasticity + optic atrophy, Leigh-like lesions	Fe-S cluster assembly factor	Mito matrix	Brain-stem / BG lesions	Peripheral neuropathy, ataxia
2p23.3	Spastic paraplegia 81	AR	618768	SELENOI	Infancy	C	Severe HSP + hypomyelination	Ethanolamine phosphotransferase-1	ER membrane	Hypomyelination + atrophy	Seizures, microcephaly
2p22.3	Spastic paraplegia 4	AD	182601	SPAST	20 – 40 y (wide)	Mainly P	Commonest AD-HSP; LL spastic weakness	Spastin AAA ATPase – microtubule severing	Cytosol / MT + ER	Mild cord atrophy (late)	Rare ataxia, distal wasting
2p13.3	Spastic paraplegia 93	AR	620938	NFU1	Infancy	C	Severe mitochondrial HSP	Fe-S cluster scaffold NFU1	Mito matrix	BG T2 + leukodystrophy	Lactic acidosis, early respiratory failure
2p11.2	Spastic paraplegia 31	AD	610250	REEP1	20 – 50 y	P (±C)	Uncomplicated HSP ± calf atrophy	ER-shaping protein REEP1	ER (motor axons)	Mild cord thinning	Distal leg wasting (mild)
2q33.1	Spastic parap	AD	605280	HSPD1	Adult	P (±C)	Gradual spastic paraparesis	Hsp60 chaperonin (protein folding)	Mito matrix	Mild brain/cord atrophy (some)	Occasional distal amyotrophy

	legia 13										
2q37.3	Spastic paraplegia 30	AD/AR	610357/ 620607	KIF1A	Childhood–teens	P (±C)	Early-onset spastic gait ± optic atrophy	Kinesin-3 motor for axonal transport	Axonal microtubules	Thin corpus callosum in some	ID, seizures (variable)
3q12.2	?Spastic paraplegia 57	AR	615658	TFG	Early child	P ± C	Early HSP ± cerebellar signs	TFG – ER-to-Golgi traffic	ER-Golgi interface	Cerebellar atrophy (some)	Nystagmus
3q25.31	Spastic paraplegia 42	AD	612539	SLC33A1	Early adult	P / C	Spasticity ± distal weakness	Acetyl-CoA transporter AT-1	Golgi / ER membrane	Non-specific WM T2	Sensory loss, hearing loss (rare)
3q27-q28	Spastic paraplegia 14	AR	605229	SPG14	Adulthood	C	HSP with mild ID & distal neuropathy	–	–	Not defined	Cognitive impairment, peripheral neuropathy
4p16-p15	Spastic paraplegia 38	AD	612335	SPG38	Teens → adult	P	Rare pure HSP (mapped locus)	–	–	–	–
4p13	Spastic paraplegia 79A/ 79B	AD/AR	620221/ 615491	UCHL1	Childhood	P	Early pure HSP (HSN-SP overlap)	Neuronal de-ubiquitinase	Cytosol	Mild cord atrophy (some)	Distal sensory loss

4q25	Spastic paraplegia 56	AR	615030	CYP2U1	Childhood	P / C	Pure HSP; occasional ataxia	Cytochrome P450 2U1 (FA hydroxylase)	ER	Mild cord atrophy	Ataxia, mild ID
5q31.2	Spastic paraplegia 72A/72B	AD/AR	615625/620606	REEP2	Infancy / early child	P	Pure HSP; pes cavus, hypertonia	ER-shaping protein REEP2	ER tubules	Unremarkable	Mild tremor, jt contracture
6p25.1	Spastic paraplegia 77	AR	617046	FARS2	Childhood	C	HSP with ataxia & epilepsy	Mito Phe-tRNA synthetase	Mito matrix	Cerebellar atrophy ± BG T2	Seizures, DD
6p21.33	Spastic paraplegia 86	AR	619735	ABHD16A	Early child	C	Complex HSP + behavioral issues	PS lipase (endocannabinoid)	Microsomes / cytosol	Cerebral atrophy ± thin CC	Autism traits, pigmentary retinopathy
6q23-q24.1	Spastic paraplegia 25	AR	608220	SPG25	Adult	P	Adult-onset isolated HSP (single family)	–	–	Not reported	–
7p22.1	Spastic paraplegia 48	AR	613647	AP5Z1	Mid-adult	P (rare C)	Late-onset HSP; occasional leukodystrophy	AP-5 ζ-subunit (endosome sorting)	Late endosome / lysosome	Leukodystrophy ± thin CC	Early-onset form: dystonia, parkinsonism
7q22.1	Spastic parap	AR	612936	AP4M1	Infancy	C	AP-4 deficiency: infantile	Adaptor-protein-4 µ-subunit	Golgi / endosome	Thin CC, hypomyelination	Epilepsy, microcephaly

	legia 50						hypotonia → spasticity				
8p22	Spastic paraplegia 53	AR	614898	VPS37A	Childhood	C	HSP with cognitive impairment	ESCRT-I subunit (VPS37A)	Endosomal membrane	Leukodystrophy (severe)	Ataxia
8p21.1-q13.3	Spastic paraplegia 37	AD	611945	SPG37	Child→adult	P	Familial progressive HSP	–	–	–	–
8p11.23	Spastic paraplegia 18A/18B	AD/AR	620512/611225	ERLIN2	Early childhood	P / C	Spasticity ± seizures & contractures (juvenile PLS-like)	ER lipid-raft protein; ERAD scaffold	ER membrane	Hypomyelination in some	Developmental delay
8p11.23	Spastic paraplegia 54	AR	615033	DDHD2	Childhood	C	HSP + ID, ataxia, tremor	DDHD2 PLA1 (lipid homeostasis)	Cytosol ± Golgi	Cerebellar atrophy ± WM T2	Upper-limb tremor
8p11.21	Spastic paraplegia 85	AR	619686	RNF170	Early child	C	Spasticity + UL weakness, hearing loss	E3-ubiquitin ligase RNF170	ER membrane	Mild CB atrophy (some)	Sensorineural deafness
8q12.3	Spastic paraplegia 5A	AR	270800	CYP7B1	Juvenile–adult	P / C	Slowly progressive pure HSP; responds to chenodeoxyc	Oxysterol 7-α-hydroxylase (cholesterol catabolism)	ER membrane	Patchy WM changes in some	Optic atrophy, ataxia (variable)

							holic-acid trials				
8q24.13	Spastic paraplegia 8	AD	603563	WASHC5	Mid-adult	P	Slowly progressive gait spasticity	Strumpellin – WASH complex (actin on endosome)	Endosomal membrane	Non-specific WM change (±)	–
9p13.3	Spastic paraplegia 46	AR	614409	GBA2	Child→adult	C	Spasticity ± cerebellar ataxia, neuropathy	Non-lysosomal β-glucosidase	ER / cytosol	Cerebellar atrophy (ataxic cases)	Hepatosplenomegaly, ataxia
9q	Spastic paraplegia 19	AD	607152	SPG19	Adult	P	Late-onset slow HSP (single pedigree)	–	–	–	–
9q34.11	Spastic paraplegia 91	AD	620538	SPTAN1	Congenital → YA	C	Spasticity ± ataxia, epilepsy	α-II-spectrin (axonal scaffold)	Membrane-associated cytoskeleton	Cerebellar atrophy (ataxic cases)	Epilepsy, severe ID (early onset)
10q22.1-q24.1	Spastic paraplegia 27	AR	609041	SPG27	Childhood → early adult	P	Slowly progressive isolated spastic paraparesis	–	–	None characteristic	–
10q24.1	Spastic paraplegia	AD/AR	616586/601162	ALDH18A1	Child-teen	P (±C)	Thin habitus, HSP ± cataract	P5CS – proline biosynthesis	Mito matrix + cytosol	Mild WM change (rare)	Short stature, cataract

	9A/9B										
10q24.1	Spastic paraplegia 64	AR	615683	ENTPD1	Childhood	P	Pure childhood HSP (rare)	Ecto-ATP diphosphohydrolase 1	Plasma membrane	None characteristic	–
10q24.31	Spastic paraplegia 62	AR	615681	ERLIN1	Childhood	P (rare C)	Childhood pure HSP	Erlin-1 (ERAD complex)	ER membrane	Non-specific	–
10q24.32-q24.33	Spastic paraplegia 45	AR	613162	NT5C2	Infancy	C	Early quadriplegic spasticity + seizures	Cytosolic 5′-nucleotidase II (purine catabolism)	Cytoplasm	Global atrophy + WM loss	Microcephaly, profound ID
11p14.1-p11.2	?Spastic paraplegia 41	AD	613364	SPG41	Teen years	P	Slowly progressive spasticity	–	–	–	–
11q12.3	Silver spastic paraplegia syndrome	AD	270685	BSCL2	Early adult	P / C	“Silver syndrome”: spasticity + hand wasting	Seipin – lipid-droplet ER protein	ER membrane	Mild cord atrophy (some)	Distal UL amyotrophy
11q13.1	Spastic paraplegia 76	AR	616907	CAPN1	Adult	C	Spastic paraparesis + cerebellar ataxia	Calpain-1 protease	Cytosol → mem.	Cerebellar atrophy	Dysarthria, mild neuropathy

12q13.3	Spastic paraplegia 70	AR	620323	MARS1	Infancy	C	Early HSP + regression	Methionyl-tRNA synthetase	Cytosol	CB atrophy, WM loss	Seizures, DD
12q13.3	Spastic paraplegia 10	AD	604187	KIF5A	Child→adult	P / C	HSP ± peripheral neuropathy	Kinesin-1 heavy chain motor	Axonal micro-tubules	Cord thinning (late)	Pes cavus, ataxia
12q13.3	Spastic paraplegia 26	AR	609195	B4GALNT1	Early childhood	P	Progressive gait spasticity ± pes cavus	GM2/GD2 synthase – ganglioside biosynthesis	Golgi apparatus	Mild cerebellar atrophy (rare)	Learning disability, gaze palsy (some)
12q23-q24	Spastic paraplegia 36	AD	613096	SPG36	Teens-adult	P	Extremely rare pure HSP	–	–	–	Pes cavus
12q23.3	Spastic paraplegia 92	AR	620911	FICD	Child / teen	P	Lower-limb spastic gait	BiP AMPylase (ER stress)	ER lumen	± Periventricular WM T2	Mild cognitive / behavioural issues
12q24.31	Spastic paraplegia 55	AR	615035	MTRFR	Childhood	C	HSP with optic atrophy, neuropathy	Mito translation release factor	Mito matrix	Leigh-like brain-stem lesions	Ophthalmoplegia
13q13.3	Troyer syndrome	AR	275900	SPART	Early childhood	C	Troyer syndrome: spasticity + sh	Spartin – endosomal & lipid-droplet regulator	Cytosol & endosomes	Mild cerebral atrophy (some)	Dysarthria, hand tremor

							ort stature, distal wasting				
13q14	Spastic paraplegia 24	AR	607584	SPG24	Early childhood	P	Childhood-onset isolated HSP (very rare)	–	–	Not reported	–
13q14.2	Spastic paraplegia 88	AD	620106	KPNA3	Infancy	Mostly P	Early HSP + motor delay	Karyopherin-α3 (nuclear import)	Nucleus ↔ cytosol	Subtle WM changes (some)	Speech delay, ADHD (rare)
14q12-q21	Spastic paraplegia 32	AR	611252	SPG32	Childhood	P	Slowly progressive childhood-onset HSP	–	–	Not reported	–
14q12	Spastic paraplegia 52	AR	614067	AP4S1	Infancy	C	AP-4 syndrome variant	AP-4 σ-subunit	Golgi / endosome	Thin CC, frontal WM loss	Growth failure
14q13.1	?Spastic paraplegia 90B	AD	620417	SPTSSA	Childhood	C	HSP + speech delay & ID	Serine-palmitoyl-transferase subunit A	ER membrane	Mild brain atrophy	ADHD, mild ataxia
14q13.1	Spastic paraplegia 90A	AD	620416	SPTSSA	Childhood	P	Childhood HSP with hyper-reflexia	Serine-palmitoyl-transferase SSA	ER membrane	Typically normal	Variable severity

14q22.1	Spastic paraplegia 3A	AD	182600	ATL1	Infancy / early child	P / C	Early spastic gait, CP-like diplegia	Atlastin-1 GTPase – ER membrane fusion	Axonal ER network	Usually normal	Occasional distal amyotrophy
14q22.1	Spastic paraplegia 28	AR	609340	DDHD1	Early childhood	P / C	Childhood spastic paraparesis ± scoliosis/ataxia	Phospholipase A1 (lipid-hydrolase)	Cytosol & Golgi-associated	Mild cerebellar atrophy (some)	Scoliosis, sensorimotor neuropathy
14q24.1	Spastic paraplegia 15	AR	270700	ZFYVE26	< 10 yrs	C	HSP with thin corpus callosum, cognitive decline	FYVE-finger protein "spastizin" – endolysosomal/autophagy regulator	Endosomes & autolysosomes	Thin corpus callosum ± WM changes	Retinal dystrophy, parkinsonism
14q24.3	Spastic paraplegia 87	AR	619966	TMEM63C	Infancy	C	Early HSP, regression, seizures	Mechanosensitive ion channel	Mito + ER membranes	Progressive CB & cerebral atrophy	Visual loss, dystonia
15q11.2	Spastic paraplegia 6	AD	600363	NIPA1	Adult	P (±C)	Rapidly progressive HSP	Mg2+ transporter NIPA1	Endosome / PM	None characteristic	Rare seizures, neuropathy
15q21.1	Spastic paraplegia 11	AR	604360	SPG11	Childhood / early teens	C	HSP with thin corpus callosum and cognitive decline	Spatacsin – endolysosomal/autophagy regulator	Late endosome & lysosome	Thin corpus callosum; diffuse WM loss	Peripheral neuropathy, retinal degeneration, parkinsonism

15q21.2	Spastic paraplegia 51	AR	613744	AP4E1	Infancy	C	Same AP-4 syndrome phenotype	AP-4 ε-subunit	Golgi / endosome	Thin CC, cerebral atrophy	Feeding difficulties, seizures
15q22.31	Mast syndrome	AR	248900	ACP33	Early childhood	C	Mast syndrome: LL + UL spasticity, cognitive decline	Maspardin – Golgi/endosome trafficking protein	Golgi & endosomal membranes	Generalised cerebral white-matter atrophy	Dementia, distal amyotrophy
16p12.3	Spastic paraplegia 61	AR	615685	ARL6IP1	Infancy	C	HMSN-SP: spasticity + neuropathy	ARL6IP1 – ER-shaping protein	ER membrane	Hypomyelination; PN demyelination	Foot deformities
16q13	Spastic paraplegia 89	AR	620379	AMFR	Early child	C	Spasticity + growth failure	gp78 E3-ligase – ERAD / lipid droplets	ER membrane	Diffuse cerebral atrophy	Short stature, liver disease
16q23.1	Spastic paraplegia 35	AR	612319	FA2H	Childhood	C	HSP with demyelinating neuropathy & ataxia	Fatty-acid 2-hydroxylase (myelin glycolipid synthesis)	Endoplasmic reticulum (oligodendrocytes)	Widespread leukodystrophy / demyelination	Cognitive decline, tremor
16q24.3	Spastic paraplegia 7	AD, AR	607259	SPG7	Juvenile → adult	P / C	Slowly progressive spasticity, optic neuropathy, ± ataxia	Paraplegin – mitochondrial m-AAA protease	Mitochondrial inner membrane	Cerebellar ± brain-stem atrophy	Dysarthria, external ophthalmoplegia

17q25.3	Spastic paraplegia 82	AR	618770	PCYT2	Infancy	C	Profound DD + spasticity	CDP-ethanolamine synthase	ER / cytosol	Cerebral & CB atrophy	Nystagmus, epilepsy
19p13.2	Spastic paraplegia 39	AR	612020	PNPLA6	Childhood	C	Boucher-Neuhäuser: spasticity + ataxia, hypogonadism, chorioretinal degeneration	Neuropathy-target esterase (phospholipase)	ER membrane of neurons	Cerebellar atrophy; sometimes retinal changes	Eye-movement abnormalities, vision loss
19q12	?Spastic paraplegia 43	AR	615043	C19orf12	Childhood / teens	C	MPAN: HSP with brain-iron accumulation	Mitochondrial membrane protein (lipid / Fe-homeostasis)	Mitochondrial outer membrane	Iron deposition in globus pallidus & substantia nigra	Dystonia, parkinsonism, cognitive decline
19q13.12	Spastic paraplegia 75	AR	616680	MAG	Childhood	P	Slow progressive pure HSP	Myelin-associated glycoprotein	Myelin membrane	Generally normal	–
19q13.32	Spastic paraplegia 12	AD	604805	RTN2	Child / teen	P	Benign pure HSP	Reticulon-2 – ER tubule curvature	ER membrane	Usually normal	–
19q13.33	?Spastic paraplegia 73	AD	616282	CPT1C	Early adult	P	Pure HSP ± pes cavus	Carnitine palmitoyl-transferase 1C	Mito outer membrane	None reported	–

22q11.21	Spastic paraplegia 84	AR	619621	PI4KA	< 10 y	C	HSP + epilepsy & immunodef.	PI4-kinase-α (PI4P lipid)	Golgi / PM	Hypomyelinating leukodystrophy	Recurrent infections, IBD
Xq11.2	Spastic paraplegia 16	XLR	300266	SPG16	Early childhood	P	Familial X-linked spastic paraplegia	–	–	No distinctive pattern	–
Xq22.2	Spastic paraplegia 2	XLR	312920	PLP1	Infancy (variable)	C	Pelizaeus-Merzbacher: nystagmus → quadriplegic spasticity	Major CNS myelin proteolipid	Oligodendrocyte myelin membrane	Diffuse hypomyelination	Ataxia, hypotonia, developmental delay
Xq24-q25	Spastic paraplegia 34	XLR	300750	SPG34	Late child → early adult	P	Slowly progressive pure HSP in affected males	–	–	–	Possibly mild cord atrophy
Xq28	MASA syndrome	XLR	303350	L1CAM	Infancy / early childhood	C	MASA / L1-syndrome: spastic gait, adducted thumbs, ID	Neuronal cell-adhesion molecule L1	Axonal membrane, growth cones	Hydrocephalus or ventriculo-megaly	Severe ID, aphasia, hydrocephalus
Not Mapped	Spastic paraplegia 33	AD	610244	ZFYVE27	Adult	P	Slow progressive HSP	Protrudin – ER-endosome tether	ER & endosome	None specific	–

Supplementary Table S1 Review of the available in OMIM genes and the matching type of hereditary spastic paraplegia, together with relevant clinical information for the specific type [25]. AR – autosomal recessive. AD – autosomal dominant. XLR – X-linked

recessive. P – pure, C – complicated. HSP – hereditary spastic paraplegia. ER – endoplasmic reticulum. ID – intellectual disability. IBD – inflammatory bowel disease. CC – corpus callosum. BG – basal ganglia. CB – cerebellar.
